# A Discrete Choice Experiment on Women’s Preferences for Water Immersion During Labor and Birth: Identification, Refinement and Selection of Attributes and Levels

**DOI:** 10.3390/ijerph17061936

**Published:** 2020-03-16

**Authors:** Thomas G. Poder, Nathalie Carrier, Mathieu Roy, Chantal Camden

**Affiliations:** 1 School of Public Health, University of Montreal, Montreal, QC H3N 1X9, Canada; 2 Centre de recherche de l’Institut universitaire en santé mentale de Montréal, CIUSSS de l’Est-de-l’Île-de-Montréal, Montreal, QC H1N 3V2, Canada; 3 Centre de recherche du CHUS, CIUSSS de l’Estrie—CHUS, Sherbrooke, QC J1H 5N4, Canada; Nathalie.Carrier2@usherbrooke.ca (N.C.); Mathieu.Roy7@USherbrooke.ca (M.R.); chantal.camden@usherbrooke.ca (C.C.); 4 Health Technology and Social Services Assessment Unit, CIUSSS de l’Estrie—CHUS, Sherbrooke, QC J1H 4C4, Canada; 5 School of Rehabilitation, University of Sherbrooke, Sherbrooke, QC J1H 5N4, Canada

**Keywords:** water immersion, childbirth, preference, discrete choice experiment

## Abstract

Objectives: To identify attributes (i.e., characteristics describing a scenario) and levels (i.e., each characteristic may be defined by a different level) that would be included in a discrete choice experiment (DCE) questionnaire to evaluate women’s preferences for water immersion during labor and birth. Methods: A mixed-method approach, combining systematic reviews of the literature and patient focus groups to identify attributes and levels explaining women’s preferences. After the focus groups, preference exercises were conducted and led to the creation of the questionnaire, including the DCE. A qualitative validation of the questionnaire was conducted with women from the focus groups and with medical experts. Results: The literature reviews provided 26 attributes to be considered for childbirth in water, and focus groups identified 14 additional attributes. From these 40 attributes, preference exercises allowed us to select four for the DCE, in addition to the birth mode. Labor duration was also included, even if it was not well ranked, as it is the main clinical outcome in the literature. Validation with experts and women did not change the choice of attributes but slightly changed the levels selected. The final six attributes were: birth mode, duration of the labor phase, pain sensation, risk of severe tears in the perineum during the expulsion of the newborn, risk of death of the newborn, and general condition of the newborn (Apgar) score at 5 minutes. Conclusion: This study allowed us to detail all the stages for the design of a DCE questionnaire. To date, this is the first study of this kind in the context of women’s preferences for water immersion during labor and birth.

## 1. Introduction

Considering patient preferences is crucial for shared decision-making that promotes greater satisfaction and better clinical outcomes [1,2,3]. Indeed, knowing patients’ expectations and preferences allows physicians to collaboratively propose and select different treatment options. Healthcare institutions and decision-makers also need to know patients’ preferences in order to provide appropriate and more effective care. A well-known method used to elicit patients’ preferences is the discrete choice experiment (DCE) [4]. This allows participants to perform trade-offs between different scenarios described by attributes (i.e., characteristics describing a scenario) and levels (i.e., each characteristic may be defined by a different level). In a DCE, a series of choice cards are presented to participants. For each choice card, following an orthogonal selection procedure, levels are randomly selected for each attribute, and the respondent is asked to choose his/her favorite scenario. Doing so makes it possible to determine the relative weight of each attribute and assists in the design of future interventions.

One intervention that has gained popularity in recent years is childbirth in water [5]. Despite reported benefits, such as reduced pain and the promotion of cervical dilation, this birth mode is not traditionally included in childbirth options in public hospitals. The main criticisms of childbirth in water are the risks of the newborn inhaling water and of infection for mother and newborn. In addition, despite better clinical results, the costs of childbirth in water were greater by about CA$ 220 [6]. It is, therefore, important to explore women’s preferences to determine whether or not this mode of childbirth should be implemented in hospital settings or elsewhere.

A literature review conducted by our team leader and a more recent one published in 2018 provided similar results in regard to the benefits and risks of childbirth in water [7,8]. The level of evidence from the studies identified was low to moderate, particularly for studies analyzing the expulsion phase [7,8]. Several studies suggested that immersion during labor reduces analgesic intake [9,10,11,12,13,14] and the duration of the labor phase [9,10,12,13,15,16,17]. The Apgar score at one minute or more was the same with water birth as with land birth [13,14,18,19,20] and better at 5 min with water immersion [12,20,21]. There were no differences in maternal [12,14] or neonatal infection rates [13,14,18,19,22,23,24] and mortality [8,12,13,18,19,20,22,25]. The number of episiotomies was significantly fewer [12,13,16,17,23,25,26]; there were also fewer third and fourth degree tears, but more first and second degree tears were observed. Finally, greater maternal satisfaction [17,25,27] and relaxation, as well as less pain, were expressed by women [17,18,23]. These elements are important but do not indicate the level of importance of each one in women’s decisions to choose water immersion or not.

The objective of this study was to use a mixed-method approach to identify attributes and levels that would be included in a discrete choice experiment (DCE) questionnaire to evaluate women’s preferences for water immersion during labor and birth.

## 2. Methods

**Figure 1:**
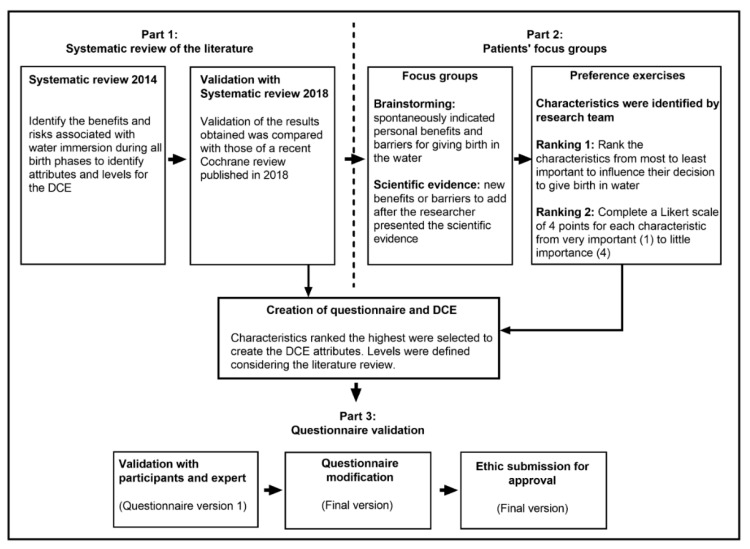
Mixed-methods approach, combining systematic reviews and patient focus groups to identify attributes and levels explaining women’s preferences for birth in water.

Using qualitative methods for the design of a DCE to define attributes and levels is highly recommended by experts [28,29,30]. In this study, we used a mixed-method approach, combining the results of two systematic literature reviews with patient focus groups, preference exercises, and one-on-one interactions with medical experts and women (Figure 1).

### 2.1. Literature Reviews

A systematic review of the literature was conducted by our team leader in 2013; details can be found elsewhere [7]. The purpose was to identify the benefits and risks associated with water immersion during all birth phases. The results of this systematic review helped to identify attributes and levels for the DCE. Since this systematic review was published in 2014, a validation of the results obtained was compared with those of a recent Cochrane review, published in 2018 [8]. 

### 2.2. Patient Focus Group

Four different focus groups took place between 26 March and 5 April 2019. The criteria to be a participant were being a woman between 18 and 45 years of age and being able to understand French. We aimed to have equal representation by having participants who were nulliparous, primiparous, multiparous and pregnant women, in addition to women who had previously experienced giving birth in water. To recruit women from various socio-economic backgrounds, participants were solicited through the network of contacts of members of the research team, through the home-birth network and through the support network for single-parent and blended families in Eastern Township, QC, Canada. Although it was a convenient sample, more efforts were produced after the recruitment of the ten first participants to recruit new participants from groups that were less represented (i.e., pregnant and with a low socio-economic background). Each focus group lasted about an hour. The objective was to determine those characteristics that have an impact on the choice of birth mode (i.e., with or without immersion in water) to define the attributes and levels for the DCE. Each focus group was conducted in three steps. First, the consent of each woman was collected, followed by a description of the aim of the study and the DCE, and the history and definition of childbirth in water. Second, brainstorming conversations took place where each woman spontaneously indicated her personal advantages or disadvantages regarding giving birth in water. Third, the researcher presented the scientific evidence from the systematic literature reviews and asked participants if they thought they might change their minds based on this new information or if they had new advantages or disadvantages to add. A discussion was also conducted at this step to identify which characteristics were important and which were not in the decision-making process of choosing a birth mode.

### 2.3. Preference Exercises

Following the focus groups, the research team identified all characteristics judged as important by women in their decision to consider water immersion during labor and birth. This was done during the analysis of the transcript of the focus groups where women clearly indicated which characteristics were important and which were not. A list of 22 characteristics/attributes (positive or negative) was established, and two ranking exercises were then proposed to the women by email. The first served to rank the 22 characteristics from one to 22 (from most to least important) that might influence their decision to give birth in water. An average for each item was calculated, and a ranking was established from smallest to largest. The second exercise was to complete a four-point Likert-type scale for each characteristic with the following values: very important (1), important (2), somewhat important (3), and of little importance (4). Two analyses were performed with these results. First, an average was identified using the Likert values and a ranking from smallest to largest was performed. Then, cumulative percentages for “very important” and “important” were identified, and the items were ranked from most important to least important.

### 2.4. Questionnaire

Considering the results from the preference exercises, the characteristics ranked the highest were selected to create the DCE attributes. Levels were defined by considering the literature review. The DCE included 12 choice cards; each card presents two scenarios according to the selected attributes and levels. For each choice card, following an orthogonal selection procedure, levels were randomly selected for each attribute. The respondent was asked to choose her preferred scenario or the option “none of these choices”. In order to test whether the responses of individuals are rational (rationality test), one of the choice cards proposed a scenario that, initially, completely dominated the other. To test the consistency of responses over time, one of the choice cards was presented twice (consistency test). A series of socio-demographic questions, definitions of childbirth in water and birth phases, experience of past childbirth, and interest in giving birth in water preceded the DCE in the questionnaire. Other measures and questionnaires designed to assess quality-adjusted life year (QALY) followed the DCE [31,32].

### 2.5. Validity of Questionnaire

The first version of the questionnaire was sent to all participants of the focus groups, a public health expert, a child development expert, a specialist in neonatal and perinatal medicine, and two general practitioners with a specialty in monitoring pregnancy. We asked them to complete the questionnaire online, to calculate the amount of time it took to complete, and to comment on the ease of completing it and on the clarity of the questions. Specifically, we asked them whether some sentences should be rephrased and if the attributes and levels selected for the DCE were appropriate. 

### 2.6. Ethics

This study was approved by the ethics committee of the CIUSSS de l’Estrie—CHUS (Comité d’éthique de la recherche of the CIUSSS de l’Estrie—CHUS # 2019-3091). All participants signed a consent form and were informed that the results would be used to design a DCE that will inform decision-makers and that they may be published in a peer-reviewed journal.

## 3. Results

All details of the process of the mixed-method approach are presented step by step.

### 3.1. Literature Reviews

The systematic review conducted by our team leader included not only randomized controlled trials, but also other designs [7]. As a result, it was more exhaustive and included more studies than the Cochrane review that was published later (i.e., 27 vs. 15) [8]. These two literature reviews identified 16 outcomes on which water immersion may have an impact during the labor phase (positive, negative or no difference from land birth), 20 for the expulsion phase, and two for the delivery of the placenta; this yielded 26 unique outcomes. For both literature reviews, the studies identified had low to moderate levels of evidence, particularly for studies analyzing the expulsion phase [7,8].

The main significant outcomes observed were the reduced duration of the labor phase with immersion [9,10,12,13,15,16,17] and the reduction of analgesic intake [9,10,11,12,13,14]. The Apgar score at one minute or more was the same [13,14,18,19,20] and better at five minutes with water immersion [12,20,21].

There is no evidence of increased adverse effects on the neonatal mortality rate [8,12,13,18,19,20,22,25] or on maternal [12,14] or neonatal infection rates [13,14,18,19,22,23,24]. With the exception of a study indicating a non-significant difference [18], no study reported the incidence of newborn death [9,12,13,19,20,25]; however, there are several where immersion was an indirect cause of death [33,34,35], the main cause being an inhalation of water resulting in death due to a lack of medical supervision. No differences were observed in regard to admission to a neonatal intensive care unit [13,14,18,19,23,24].

No differences were observed for the use of instruments during expulsion [18,19] or for caesarean section rates [18,19]. The number of episiotomies required was found to be significantly lower when the newborn was expelled into the water [12,13,16,17,23,25,26]. There were fewer third and fourth degree tears with immersion in water but more first and second degree tears. In addition, several studies showed that water immersion resulted in more perinea remaining intact [12,13,20]. 

Finally, women who have experienced labor and delivery in water are mostly very satisfied and wish to repeat it for a future birth [17,25,27]. In addition, some studies indicated that women experienced less pain during water birth [17,18,23,27]. 

### 3.2. Focus Groups

The focus groups included 17 women between 24 and 54 years old. Although one of the inclusion criteria was for participants to be between 18 and 45 years old, one was 54 years old. It is important to note that this older participant took part only in the focus group and not in the preference exercises. One group took place during a planned activity in the support group for single-parent and blended families. As the group was open to all women present, no one was excluded (including the older participant mentioned above). Three other focus groups took place in our hospital. Among the participants were three childless women, seven primiparous women including two who were pregnant, and seven multiparous women. Water immersion had been experienced by four of these participants, one at her home and three at a birthing house. In one of these cases, a woman had a hemorrhage while pulling on the umbilical cord, but she had no complications and did not require transfer to the hospital. The hemorrhage stopped when she emerged from the water. In all cases, the women were very satisfied with their experience.

The first focus group was planned for five participants (Fleurimont), but only two attended. The second included seven women (Youville), the third had four women (community center) and, finally, the fourth group comprised four participants at the same site as the first focus group and included two participants who were unable to attend the first focus group. 

A total of 22 important characteristics identified as such by participants emerged from the brainstorming conversations, eight from the literature reviews, and 14 from the participants (Table 1). The characteristics have been grouped into four main categories: (1) impact for the mother (i.e., pain reduction, reduction of perineal injuries, reduction of the duration of active labor phase); (2) impact for the newborn (i.e., mortality risk for newborn is identical, general condition of newborn slightly better (Apgar), risk of infection, risk of inhalation of water by newborn); (3) water effect (i.e., calming/relaxing, less pressure felt on the abdomen, etc.); (4) birthing house (i.e., distance between birthing house and hospital in case of complications, environment less medical than in the hospital).

### 3.3. Preference Exercises

**Figure 2:**
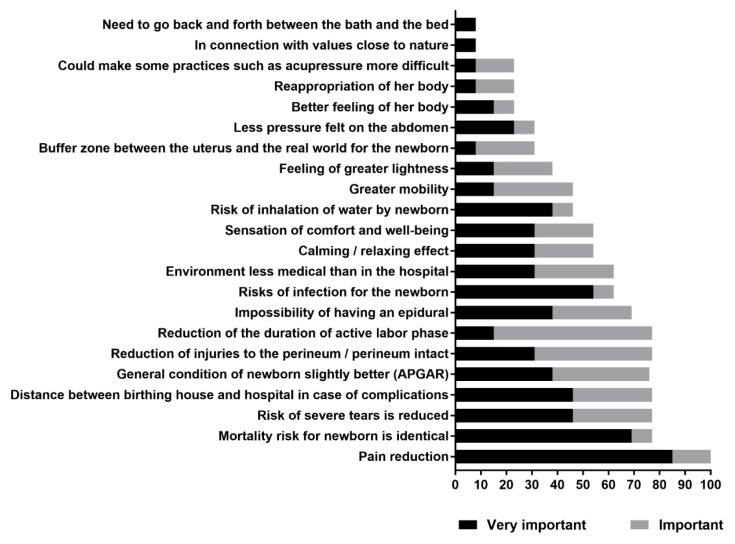
Classification of characteristics according to their importance for choosing to give birth in water.

The results of the preference exercises are provided in Figure 2 and Table 2. Results from the Likert-type scale for “very important” and “important” are presented in Figure 2. All women considered pain reduction as very important (84.6%) or important (15.4%) in the decision to choose water immersion. Other important characteristics were the mortality risk for the newborn, which is identical, and the risk of severe perineal tears, which is reduced. The ranking of characteristics that may influence a woman’s choice to give birth in the water according to the three ranking methods are presented in Table 2. The most important characteristics were very similar for all three methods of ranking. 

Considering that having more than seven attributes in a DCE is not recommended, the decision was made to keep the five most-preferred characteristics in addition to the birth mode. At first, it was decided to keep the attributes that were ranked in the top three among the three ranking results at least once, i.e., pain reduction, mortality risk of newborn is identical, risk of severe perineal tears is reduced, perineal injuries are reduced, and perineum remains intact. Since the last two characteristics were redundant, only one was kept (i.e., risk of severe tears is reduced). We then decided to keep the attribute that was ranked at least twice in the top five among the three ranking results, i.e., general condition of newborn slightly better (Apgar score). Finally, we also included active labor duration, even if it was ranked only seventh, since it is the clinical outcome most frequently reported in the literature, indicating a significant difference from land birth [9,10,12,13,15,16,17].

### 3.4. Validity of the Questionnaire

The validation with medical experts (i.e., one specialist in neonatal and perinatal medicine and two general practitioners) allowed us to better define conventional birth and water birth. They suggested several changes for the levels associated with the Apgar score in the DCE, as well as including a question asking the respondent who she would like to monitor her pregnancy (i.e., midwife or doctor) and where she may prefer to give birth (i.e., hospital, birthing house, home). Women from the focus groups and other experts allowed the refining of several questions (marital status, occupation, education, annual income, interest in giving birth in the water, the addition of two techniques in the methods used during previous childbirth, i.e., exercise ball and acupressure). For women who had previously experienced a water birth, a question asking whether or not they needed medical assistance was added. Following the questioning of two pregnant women for the SF-6Dv2 (an instrument used to calculate quality-adjusted life year), a sentence was added indicating that pregnant women should describe their current health status and not their usual health status. 

For the first choice card in the DCE, one of the scenarios (the one with water birth) completely dominated the other (conventional birth) as it was initially set to be a rationality test. However, one woman thought that this forced the respondent to choose water birth even if it was not her preference. Therefore, we changed the first choice card to have the same levels (best one) for all attributes, except for the birth mode where, in scenario one, the conventional birth was presented and, in scenario two, the birth corresponding to water immersion in all phases (labor, expulsion, and delivery of the placenta) was presented. 

As many aspects of water birth could hardly be part of the DCE (e.g., distance between birthing house and hospital in case of complications, calming/relaxing effect, sensation of comfort and well-being), a list of characteristics that might either encourage or discourage women from giving birth in water was added to the questionnaire. These characteristics were classified with a four-point Likert-type scale as in the preference exercise previously described: very important (1), important (2), somewhat important (3), and of little importance (4). Some participants found the DCE exercise difficult, but, in general, they found the questionnaire clear and concise, and noted that the choice of attributes and levels reflected the discussions in the focus groups well. The final selection of attributes and levels is described in Table 3.

## 4. Discussion

The combination of literature reviews, focus groups, preference exercises, and comments from participants and medical experts aided in the selection of the most appropriate attributes and levels for the DCE questionnaire. These results will help to elicit women’s preferences for water birth within a larger DCE study to be conducted. The next step will be to conduct a survey using a large sample of women of childbearing age to define their preferences for water birth in order to inform healthcare institutions whether or not they should adapt their services to provide this mode of labor and delivery. Indeed, this information about women’s preferences is needed since there may be some barriers to implement water immersion in hospitals (e.g., logistics and medical culture) [5,7,27], although the extra costs may be considered relatively small as compared to the expected benefits [6].

To the best of our knowledge, no study has used a DCE method to evaluate women’s childbirth preferences by considering the aspect of immersion in water. Larkin et al. used mixed methods with qualitative analysis and DCE to assess women’s preferences for birth, but did not consider water immersion [36]. The most important attributes included in the study by Larkin et al. were the availability of pain relief, partnership with the midwife, and individualized care. 

In our study, the three main characteristics of interest in choosing or declining water birth were pain reduction; the mortality risk of the newborn, which was identical; and the risk of severe perineal tears, which was reduced. Several studies have shown a reduction in analgesic intake with water immersion [9,10,11,12,13,14], and some women have mentioned a reduction in pain during water birth [17,18,23]. Regarding reduction in perineal tears, some studies have observed a decrease in third and fourth degree tears. In terms of mortality, the results are less clear. The studies identified have not been able to demonstrate statistically significant differences [8,12,13,18,19,20,25], but whether this is because there are no differences or because the studies are too small is unclear. However, several cohort studies with a large number of women (several thousands), such as Geissbuehler et al. [12], suggest that there are no differences between these two birth modes. 

A reduction in the duration of the labor phase was considered in the attributes, although it did not emerge from the results. As this is one of the main effects observed in the literature, it was decided, with the experts, to add this aspect. With a larger sample, we will be able to determine whether or not this aspect is important during the survey.

One limitation was that several potential attributes are related only to giving birth in water and are less applicable to the DCE that compares different alternatives (i.e., land birth and water birth). For example, we have seen that the distance between the hospital and the birthing house can reduce interest in giving birth in the water. In Quebec, only one hospital offers the possibility of delivery in water. Elsewhere, this mode is offered only at home or in a birthing house. In our region, there is a distance of 13 km between the birthing house and the hospital, which reduces interest in choosing this birth mode. Women in focus groups defined the majority of these attributes, which have also been observed in the literature but not measured. To compensate for the inability to include several attributes in the DCE, specific questions using Likert-type scales were added to the questionnaire to assess whether these characteristics would have a positive or negative impact on a woman’s choice to give birth in water. This additional information may complement the results of the DCE and possibly allow for the adaptation of services offered to women in hospitals. However, future research may be needed to specifically focus on these aspects, as well as on the barriers and facilitators that may guide the hospital decision-makers to implement water birth (or not) in specialized birth units.

A strength of this study is the fact that we were able to collect the opinions of respondents including nulliparous, primiparous, multiparous and pregnant women, as well as women who had previously experienced giving birth in water. However, to be more representative, we would have liked to have more pregnant women in our sample. In addition, despite the diversity of women interviewed, it was still relatively easy to inquire about and obtain women’s preferences. In contrast, one limitation was that, in some very diverse focus groups, several women who had not had children did not feel comfortable about giving their opinions on conventional or water birth, especially in the presence of women who had experienced water birth. Another limitation was having to conduct several focus groups, since some groups were smaller than expected. Despite these limitations, from one focus group to another, the same preferences were usually highlighted, except in the group with women who had already experienced of giving birth in water, which further enriched the discussion. In addition, our selection of attributes is consistent with what was found in the literature.

## 5. Conclusions

This study enabled the detailing of all the stages necessary to realize the design of a DCE questionnaire, from the process of identification through to the final choice of the attributes and levels. This is also, to the best of our knowledge, the first study to address the preferences of women for water birth. It will be interesting to analyze women’s preferences on a larger scale with the survey. These results will collect and elucidate women’s interests in two major birth modes and will help formulate recommendations to health care institutions, especially in Quebec. As such, implications of this study may support the need for a more patient-centered approach of care, as well as to show evidence in favor of alternatives initiated by patients and citizens [37].

## Figures and Tables

**Table 1:** List of characteristics identified during focus groups.

Characteristics
Impact for the mother
Pain reduction
Risk of severe tears is reduced
Reduction of injuries to the perineum/perineum intact
Reduction of the duration of active labor phase
Impossibility of having an epidural ^1^
Impact for the newborn
Mortality risk for newborn is identical
General condition of newborn slightly better (Apgar)
Risk of infection for the newborn
Risk of inhalation of water by newborn
Water effect (zen, soothing, etc.)
Calming/relaxing effect ^1^
Sensation of comfort and well-being ^1^
Less pressure felt on the abdomen ^1^
Greater mobility ^1^
Better feeling of her body ^1^
Feeling of greater lightness ^1^
Reappropriation of her body ^1^
Buffer zone between the uterus and the real world for the newborn ^1^
In connection with values close to nature ^1^
Could make some practices such as acupressure more difficult ^1^
Need to go back and forth between the bath and the bed ^1^
Birthing House
Distance between birthing house and hospital in case of complications ^1^
Environment less medical than in the hospital ^1^

^1^ New characteristics were defined by women, others come from the literature.

**Table 2:** Ranking of characteristics to make the choice to give birth in the water.

Characteristics	Score Likert	% Important	Ranking
Pain reduction	1	1	1
Mortality risk for newborn is identical	2	2	8
Risk of severe perineal tears is reduced	3	3	2
Reduction of injuries to the perineum / perineum intact	5	6	3
General condition of newborn slightly better (Apgar)	4	5	6
Distance between birthing house and hospital in case of complications	6	4	11
Risk of infection for the newborn	7	9	9
Reduction of the duration of active labor phase	8	7	7
Calming/relaxing effect	9	11	4
Impossibility of having an epidural	10	8	14
Environment less medical than in the hospital	11	10	13
Risk of inhalation of water by newborn	12	13	12
Sensation of comfort and well-being	13	12	5
Less pressure felt on the abdomen	14	17	10
Greater mobility	15	14	15
Better feeling of her body	16	18	18
Feeling of greater lightness	17	15	16
Reappropriation of her body	18	19	17
Buffer zone between the uterus and the real world for the newborn	19	16	19
Could make some practices such as acupressure more difficult	20	20	22
Need to go back and forth between the bath and the bed	21	22	20
In connection with values close to nature	22	21	21

Notes: Yellow color is for ranking 1–3; Pink color for ranking 4–5; Blue color for ranking 6–22.

**Table 3:** Attributes and levels for the discrete choice experiment (DCE).

Attribute	Levels
Birth mode	Conventional birth (land birth) Water immersion (labor phase only) Water immersion (labor phase and expulsion of the newborn) Water immersion (labor phase, expulsion of the newborn and delivery of the placenta)
Duration of the labor phase	3 h 5 h 7 h
Pain sensation	Low Moderate High
Risk of severe tears in the perineum (muscular assembly that extends from the pubis to the coccyx) during the expulsion of the newborn	Very low risk (1–2%) Low risk (3–4%)
Risk of death of the newborn	0.8 deaths per 1000 live births 1.2 deaths per 1000 live births 4.6 deaths per 1000 live births
General condition of the newborn (Apgar score at 5 minutes)	Very good physical condition (score of 10/10) Good physical condition (score of 8/10) Average physical condition (score of 6/10)

## Data Availability

Data are presented in the manuscript.

## Abbreviations

Canadian dollar (CA$); discrete choice experiment (DCE); quality-adjusted life-year (QALY).
